# Imported Dengue Hemorrhagic Fever, Europe

**DOI:** 10.3201/eid1408.080068

**Published:** 2008-08

**Authors:** María Jesús Pinazo Delgado, José Muñoz Gutierrez, Ljiljana Betica Radic, Tomislav Maretic, Sime Zekan, Tatjana Avšič-Županc, Ethel Sequeira Aymar, Antoni Trilla, Joaquim Gascon Brustenga

**Affiliations:** * Barcelona International Health Research Center, Barcelona, Spain; † August Pi Sunyer Biomedical Research Institute, Barcelona; ‡ University of Barcelona Hospital Clinic, Barcelona; § General Hospital, Dubrovnik, Croatia; ¶ University Hospital for Infectious Diseases Dr. Fran Mihaljevic, Zagreb, Croatia; # Institute of Microbiology and Immunology, Ljubljana, Slovenia; ** Centro de Atención Primaria del Sector del Eixample (CAPSE), Barcelona

**Keywords:** Dengue hemorrhagic fever, imported, Europe, letter

**To the Editor:** Dengue infection is an endemic and epidemic urban disease (*1*), transmitted by infected *Aedes* mosquitoes. Its incidence is increasing in tropical and subtropical areas (*1*,*2*) because of 1) introduction of the virus into areas where it was not previously endemic, and 2) the spread of the 4 serotypes and the vector in disease-endemic areas (*2*,*3*). Infection with 1 serotype provides lifelong homologous immunity only for that serotype, and after a few months, the presence of nonneutralizing antibodies increases the risk for progression to dengue hemorrhagic fever (DHF) or dengue shock syndrome when the patient is infected by any of the other 3 serotypes (*3*,*4*). We report an imported case with severe clinical manifestations that fulfills DHF criteria (*5*).

A 33-year-old Spanish woman who had worked in Anantapur, India, for 180 days, returned to Spain on August 1, 2007; on August 3, she traveled to Dubrovnik, Croatia, on holiday. She also had visited Thailand 45 days before August 1 and Brazil 2 years ago. Two months previously, she experienced a 3-day episode of fever that spontaneously resolved but without laboratory evidence of dengue. On August 6, she exhibited a high fever, chills, headache, arthralgia, and myalgia, with hypotension and was admitted to the hospital. Three days later, a confluent maculopapular rash developed. Dubrovnik hospital laboratory values were hemoglobin (Hb) 143 g/L, packed cell volume (PCV) 41.6%, mean corpuscular volume (MCV) 84.6 fL, platelet count 97 × 10^9^/L, leukocyte count 1.96 × 10^9^/L, aspartate aminotransferase (AST) 45 U/L, alanine aminotransferase (ALT) 31 U/L, AP 73 U/L, and lactate dehydrogenase (LDH) 198 U/L. On the fifth day of illness, platelet count was 50 × 10^9^/L. Because viral hemorrhagic fever was suspected, the patient was referred to a specialized hospital in Zagreb. Chest radiograph and abdominal ultrasound scan showed bilateral pleural and peritoneal effusions.

The patient was treated with fluid and plasma replacement, antipyretics, and ceftriaxone plus doxycycline to counteract bacterial and other possible tick-borne infections. She was placed under strict isolation measures while awaiting final diagnosis. The patient was transferred to Barcelona (Spain) University Hospital on August 14; on the basis of her clinical symptoms, hemorragic fever was suspected. She exhibited headache, arthralgia, and myalgia. The fever subsided 9 days after the onset of symptoms. Clinical examination showed a maculopapular rash involving the face, thorax, limbs, and palms and soles, with diffuse petechiae and bruising (Figure). Barcelona University Hospital laboratory values were Hb 105 g/L, PCV 32%, MCV 86, prothrombin time 12.4 s, AST 347 U/L, ALT 322 U/L, gamma-glutamyl transferase 114 U/L, alkaline phosphatase 194 U/L, LDH 544 U/L, bilirubin 0.5 mg/dL, and C-reactive protein level 6.93 mg/dL. Platelet count and renal function were within normal limits. Urine, blood, and stool cultures were all negative for bacterial infections.

**Figure:**
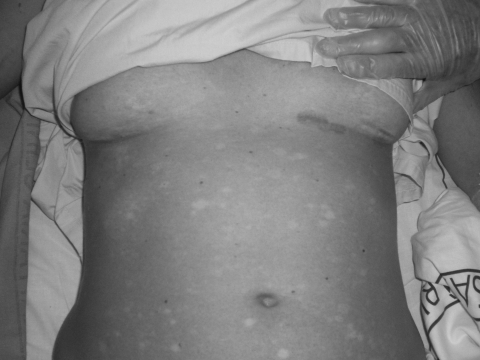
Maculopapular rash with diffuse petechiae, with areas of normal skin and bruising under the breast.

Serologic tests on day 3 and day 11 after the onset of symptoms were not reactive for Crimea-Congo hemorrhagic fever (CCHF), chikungunya, yellow fever, Hantaan, Puumala, and Dobrava viruses; HIV 1 and 2; parvovirus B19; cytomegalovirus; Epstein-Barr virus; or rickettsial diseases. Immunoglobulin (Ig) M tests on day 3 for all 4 dengue virus serotypes were negative. Positive IgG were 1:320 (type 1) and 1:100 (type 3 and 4). A second sample on day 11 showed all 4 IgG serotypes >1:10,000, and IgM >1:10,000 for serotypes 1, 2, and 4. Results of real-time PCR for CCHF were negative but reverse transcription–PCR multiplex for dengue virus was positive for dengue type 1 virus. The patient recovered and was monitored for 2 months.

Since 1977, 15 cases of imported DHF have been reported in Europe (*6*,*7*). The 4 World Health Organization (WHO) criteria for DHF diagnosis are 1) fever related to the current process, 2) hemorrhagic manifestations, 3) low levels of platelets (<100 × 10^9^/L) and 4) increased capillary permeability (*5*). Our patient fulfilled all 4 criteria. Few cases of reported DHF fulfill criterion 3 due to the short duration of severe thrombocytopenia in mild clinical forms (*8*). Increased vascular permeability was shown in our patient by the peritoneal and bilateral pleural effusions.

The probability of diagnosing dengue fever in Europe increases with travel to dengue-endemic areas, in view of the increase of DHF numbers (2006–2007) and several outbreaks around the world, even during the nondengue season (*9*). Frequent travelers are more at risk for DHF. In a recent European publication, 17% of patients with imported dengue fever exhibited a secondary immune response, thus having a higher risk of developing DHF in the future (*6*). Serologic tests confirm dengue infection only if a 4-fold increase in titers in consecutive serum samples occurs, as in our case.

In dengue-endemic areas, despite the higher disease incidence, many cases still fail to meet WHO criteria (*9*). A comprehensive revision of dengue and DHF series (*8*) shows differences in applying WHO criteria for diagnosis, and sometimes the correlation was poor between criteria-fulfilling cases and severity of disease. Some reports (*6*,*8*) suggest that WHO criteria should be reviewed and perhaps new parameters should be established to define severe dengue disease.

Although our patient was not infected in Europe, lessons from the recently described chikungunya outbreak in Italy indicate the possibility of new arbovirus outbreaks in previously non–disease-endemic areas due to the increasingly established presence of vectors like *Ae. albopictus* (*10*).

Dengue virus infection should therefore be considered in the differential diagnosis of fever in returning travelers. DHF diagnosis, although unusual, could become more frequent in the future.
