# *Erratum:* Vol. 14 No. 8

**DOI:** 10.3201/eid1508.999001

**Published:** 2009-08

The author list was incorrect in the article Imported Dengue Hemorrhagic Fever, Europe (M.J. Pinazo et al.). The authors were María Jesús Pinazo, José Muñoz, Ljiljana Betica, Tomislav Maretic, Sime Zekan, Tatjana Avsic-Zupanc, Ethel Sequeira, Antoni Trilla, and Joaquim Gascon. The article has been corrected online (www.cdc.gov/eid/content/14/8/1329.htm).

